# Producing High-Accuracy Lattice Models from Protein Atomic Coordinates Including Side Chains

**DOI:** 10.1155/2012/148045

**Published:** 2012-08-15

**Authors:** Martin Mann, Rhodri Saunders, Cameron Smith, Rolf Backofen, Charlotte M. Deane

**Affiliations:** ^1^Bioinformatics, University of Freiburg, Georges-Köhler Allee 106, 79110 Freiburg im Breisgau, Germany; ^2^Theoretical Biochemistry, University of Vienna, Währingerstraße 17, 1090 Vienna, Austria; ^3^Department of Statistics, Oxford University, 1 South Parks Road, Oxford OX1 3TG, UK

## Abstract

Lattice models are a common abstraction used in the study of protein structure, folding, and refinement. They are advantageous because the discretisation of space can make extensive protein evaluations computationally feasible. Various approaches to the protein chain lattice fitting problem have been suggested but only a single backbone-only tool is available currently. We introduce LatFit, a new tool to produce high-accuracy lattice protein models. It generates both backbone-only and backbone-side-chain models in any user defined lattice. LatFit implements a new distance RMSD-optimisation fitting procedure in addition to the known coordinate RMSD method. We tested LatFit's accuracy and speed using a large nonredundant set of high resolution proteins (SCOP database) on three commonly used lattices: 3D cubic, face-centred cubic, and knight's walk. Fitting speed compared favourably to other methods and both backbone-only and backbone-side-chain models show low deviation from the original data (*~*1.5 Å RMSD in the FCC lattice). To our knowledge this represents the first comprehensive study of lattice quality for on-lattice protein models including side chains while LatFit is the only available tool for such models.

## 1. Introduction

It is not always computationally feasible to undertake protein structure studies using full atom representations. The challenge is to reduce complexity while maintaining detail [1–3]. Lattice protein models are often used to achieve this but in general only the protein backbone or the amino acid centre of mass is represented [4–12]. A huge variety of lattices and energy functions have previously been developed and applied [4, 13, 14].

In order to evaluate the applicability of different lattices and to enable the transformation of real protein structures into lattice models, a representative lattice protein structure has to be calculated. Ma$\overset{˘}{\text{n}}$uch and Gaur have shown the NP completeness of this problem for backbone-only models in the 3D-cubic lattice and named it the *protein chain lattice fitting (PCLF) problem* [15].

The PCLF problem has been widely studied for backbone-only models [13, 16–24]. The most important aspects in producing lattice protein models with a low root mean squared deviation (RMSD) are the lattice coordination number and the neighbourhood vector angles [18, 23]. Lattices with intermediate coordination numbers, such as the face-centred cubic (FCC) lattice, can produce high resolution backbone models [18] and have been used in many protein structure studies (e.g., [3, 25, 26]). However, the use of backbone models is limited since they do not account for the space required for side chain packing.

To overcome this restriction lattice protein models that include side chains have been introduced [27–33]. Reva et al. [32] have, to our knowledge, developed the only previous approach to solve the PCLF problem including side chains. They apply dynamic programming to find an optimal solution according to their error function. Unfortunately, the approach is shown to often yield no solution in the 3D cubic lattice. The CABS tools by Kolinski and coworkers utilize a hybrid on-lattice (backbone) and off-lattice (side chain) protein representation to study folding dynamics but do not attempt to answer the PCLF problem [31, 34].

In this paper we use the side chain model definition of Bromberg and Dill [28], where each amino acid is represented by two on-lattice monomers: one represents the side chain and one the *C*_*α*_ atom. This explicit representation of side chains prevents unnatural collapse during structural studies [35] and enables the reconstruction of full atom protein data [36]. Full on-lattice protein models are constrained in their possible side chain placement but enable exhaustive studies of folding kinetics and structure space [11, 37, 38] not applicable within off-lattice side chain models like the CABS approach.

To the best of our knowledge, there is only one other publicly available implemented approach, namely, L ocalMove, to derive lattice protein models from real proteins despite a large number of published methods. LocalMove is a web interface introduced by Ponty et al. [22] for backbone-only models in 3D-cubic and FCC lattice and applies a Monte-Carlo search in order to find lattice protein models.

**Figure 1:**
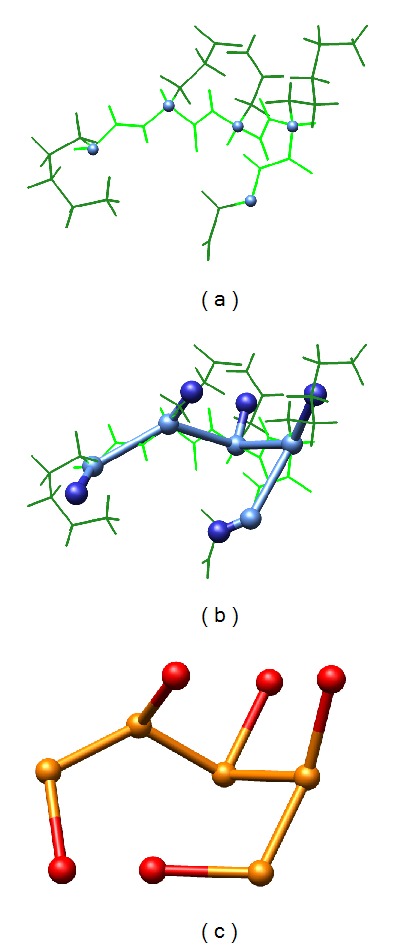
The diagram depicts the fitting process of LatFit for side chain models. (a) Original full atom data is given. The five *C*_*α*_ atoms of the segment are highlighted as balls while the backbone and side chain parts are given in light and dark green, respectively. (b) The coordinates for each amino acid to fit are extracted, that is, for side chain models the *C*_*α*_ position (light blue) and the centroid of the side chain (dark blue). (c) These positions are fitted to derive an according lattice protein model in the underlying lattice (here 3D knight's walk lattice).

We present our tool LatFit to tackle this lack of available implementations. The program is freely available for academic download and as a webserver: http://cpsp.informatik.uni-freiburg.de/LatFit/. LatFit solves the PCLF problem, that is, transforms a protein from full atom coordinate data to a lattice model, and is available as both a stand-alone tool for high-throughput pipelines and a web interface for *ad hoc* usage. A new fitting procedure that optimises distance RMSD enables rotation-independent lattice model creation of protein structures. The method is applicable to arbitrary lattices and handles both backbone and side chain representations with equivalent accuracy. A depiction of the workflow is given in Figure 1.

Utilising LatFit we present the first comprehensive study of lattice quality for protein models including side chains. In our test, LatFit fitted the majority of models on an FCC lattice within 1.5 Å RMSD.

## 2. Material and Methods

In order to enable a precise formulation of the method we introduce some preliminary definitions. A lattice *L* is a set of 3D coordinates *x* defined by a set of neighboring vectors *υ* ∈ *N*. The neighboring vectors are of equal length (∀_*υ*,*υ*′∈*N*_:|*υ* | = |*υ*′|), each with a reverse within the neighborhood (∀_*υ*∈*N*_ : −*υ* ∈ *N*), such that each coordinate in *L* can be expressed by a linear combination of the neighboring vectors, that is, *L* = {  *x* | *x* = ∑_*υ*∈*N*_ *d* · *υ*∧*d* ∈ *ℤ*_0_^+^}. |*N*| gives the coordinate number of the lattice, for example, 6 for 3D-cubic or 12 for the FCC lattice.

A lattice protein structure with side chains of length *l* is defined by a sequence of lattice nodes *M* ^*b*^ = (*M*_1_^*b*^,…, *M*_*l*_^*b*^) ∈ *L* ^*l*^ representing the backbone monomers of the protein (one for each amino acid) and the according sequence *M* ^*s*^ = (*M*_1_^*s*^,…, *M*_*l*_^*s*^) ∈ *L* ^*l*^ for the side chain positions. A valid structure ensures backbone connectivity (∀_*i*<*l*_ : *M*_*i*_^*b*^ − *M*_*i*+1_^*b*^ ∈ *N*), side chain connectivity (∀_*i*_ : *M*_*i*_^*b*^ − *M*_*i*_^*s*^ ∈ *N*), as well as self-avoidance (∀_*i*≠*j*_ : *M*_*i*_^*b*^ ≠ *M*_*j*_^*b*^∧*M*_*i*_^*s*^ ≠ *M*_*j*_^*s*^ and ∀_*i*,*j*_ : *M*_*i*_^*b*^ ≠ *M*_*j*_^*s*^). The two sets together define the lattice protein structure *M* = (*M* ^*b*^, *M* ^*s*^).

### 2.1. Fitting Procedure

Given a protein structure of length *l* in Protein Database (PDB) format [39], LatFit builds up the lattice protein sequentially, one amino acid at a time, starting from the amino terminus.

First, all neighboring vectors *υ* ∈ *N* of the used lattice *L* are scaled to a length of 3.8 Å, which is the mean distance between consecutive *C*_*α*_ atoms and close to the mean distance between a *C*_*α*_ atom and the associated side chain centroid. The latter distance was found to be on average *≈*3.6 Å within available PDB structures (data not shown). While this ignores the shorter CIS-PRO *C*_*α*_ linkage and the nonexistence of a side chain for Glycine, this scaling enables a reasonable mapping of proteins into the lattice, where each amino acid will be represented by two monomers and all covalent bonds are scaled to |*υ* | = 3.8 Å. Therefore, all resulting measures will be directly interpretable in Å units.

The positions for each amino acid *i* to be fitted, that is, the *C*_*α*_ position of the backbone *P*_*i*_^*b*^, and the centroid *P*_*i*_^*s*^ (geometric center) of all nonhydrogen atom coordinates of the side chain, are extracted from the PDB file. They form the data to fit *P* = (*P* ^*b*^, *P* ^*s*^).

The lattice model is derived by one of the following procedures optimising either a distance or coordinate RMSD. Both methods are introduced for lattice proteins including side chains but can be used to derive backbone-only lattice models as well. A sketch of the fitting workflow is given in Figure 1.

### 2.2. dRMSD Optimisation

The fitting follows a greedy iterative chain-growth procedure. The initial lattice model's backbone and side chain position (*M*_1_^*b*^ and *M*_1_^*s*^) are placed arbitrarily but adjacent (*M*_1_^*b*^ − *M*_1_^*s*^ ∈ *N*). For each iteration 1 < *i* ≤ *l*, all valid placements of the next *M*_*i*_^*b*^ and *M*_*i*_^*s*^ on the lattice are calculated. A distance RMSD (dRMSD, Eqn. 1) evaluation is used to identify the best *n*_keep_ structures of length *i* for the next extension iteration. Since dRMSD is a rotation/reflection-independent measure, symmetric structures must be filtered.

To calculate the final fit of the initial protein *P*, a superpositioning of the dRMSD-optimised structure *M* and a reflected version *M*′ is done using the method by Kabsch [40]. The superpositioning translates and rotates *M*/*M* ^′  ^in order to achieve the best mapping onto *P*. The superpositioning with lowest coordinate RMSD (cRMSD, (2)) is selected and finally returned.

(1) dRMSD=∑i<j(|Pi−Pj|−|Mi−Mj|)2l·((2·l)−1)with  P=Ps∪Pb,  and  M=Ms∪Mb.

(2) cRMSD=∑i=1l(|Pib−Mib|)2+(|Pis−Mis|)22·l.

### 2.3. cRMSD Optimisation

A cRMSD evaluation according to (2) depends on the superpositioning of the protein and its model. Thus, the best relative lattice orientation has to be identified in addition to the best model. Once the orientation is fixed, a cRMSD evaluation allows for a fast, additive RMSD update along the chain extension.

We implement a cRMSD-optimising method following [6, 18] as an alternative fitting strategy. In general a user defined number of rotation intervals *r* are performed for each of the XYZ rotation axes. For each rotation, we transform *P* ^*b*^ and *P* ^*s*^ into ${\hat{P}}^{b}$ and ${\hat{P}}^{s}$, respectively, to obtain the rotated current target structure.

The fitting procedure follows a chain-growth approach: *P*_1_^*b*^ is placed onto an arbitrary lattice node *M*_1_^*b*^. The according side chain monomer *M*_1_^*b*^ is placed to the adjacent node closest to the position *P*_1_^*s*^ to be represented. Now, all valid placements of the next *M*_*i*_^*b*^ and *M*_*i*_^*s*^ on the lattice are calculated. Using the coordinate RMSD (cRMSD, (2)) we evaluate all derived models and keep the best *n*_keep_ for the next extension following [18] until all amino acids have been placed.

By applying the above cRMSD-based fitting procedure we obtain the best fit for the current rotation. An iterative application of this procedure then results in the overall best fit for all screened rotations. Since our screen of XYZ rotations was discretised, the current rotation might be refineable. Therefore, another rotational refinement can be applied that investigates *r* ^ref^ small rotation intervals around the best rotation from the first screen [6].

The run time of the cRMSD-method scales with respect to the lattice coordination number, *n*_keep_, and most importantly the number of rotation intervals *r* and *r* ^ref^ considered.

### 2.4. Further Features

Coordinate data in the PDB is often incomplete. For example flexible loop structures are hard to resolve by current methods [41]. This results in missing coordinate data for certain substructures within PDB files. LatFit enables a structural fitting of even such fragmented PDB structures and produces a lattice protein fragment for each fragment of the original protein.

Currently, LatFit supports the 2D-square, 3D-cubic (CUB, 100), 3D-face centered cubic (FCC, 110), and 3D knights walk (210) lattice. The modular software design of our open source program enables an easy and straight forward implementation of other lattices via a specification of the according neighboring vectors *N*.

The implementation is open source and freely available for academic use at http://www.bioinf.uni-freiburg.de/Software/LatPack/.

### 2.5. Webserver

The web interface of LatFit, integrated into the CPSP web tools [42], enables *ad hoc* usage of the tool. Either a protein structure in PDB format can be uploaded or a valid identifier from the PDB database given. In the latter case, the full atom data is automatically retrieved from the database.

Our default parameters enable a direct application of LatFit resulting in a balanced tradeoff between runtime and fitting quality. The computations are done remotely on a computation cluster while the user can trace the processing status via the provided job identifier and according link. Results are available and stored for 30 days after production.

Supported output formats of LatFit are the PDB format, the Chemical Markup Language (CML) format, as well as a simple XYZ coordinate output. The output files are available for download. In addition, a highly compact string representation of the lattice protein is also given in absolute move strings that encode the series of neighboring vectors *υ* ∈ *N* along the structure.

The generated absolute move string can be directly used to apply other lattice protein tools onto the resulting structures, for example, from the CPSP package for HP-type lattice protein models [10, 42] or from the LatPack tools for arbitrary lattice models [11, 38].

**Figure 2:**
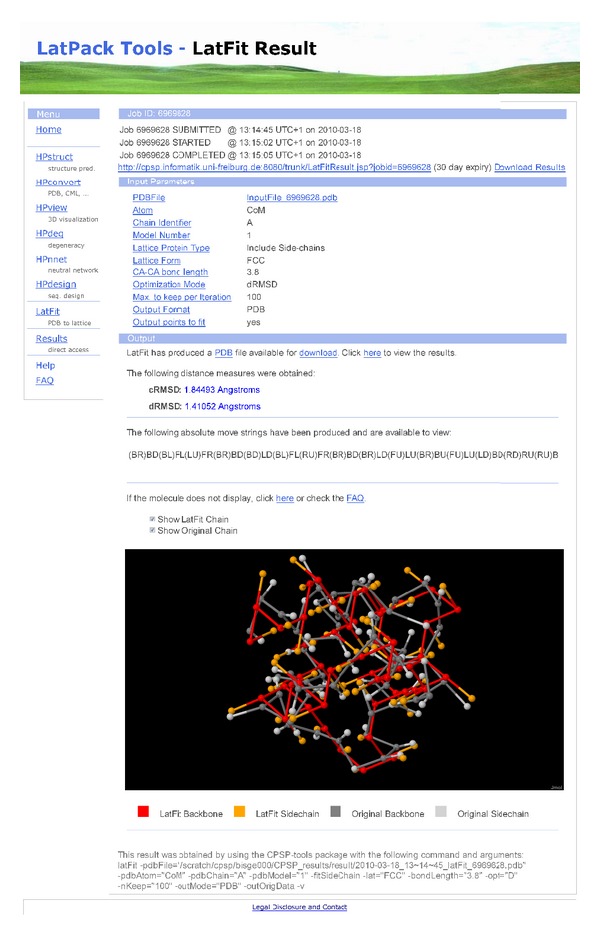
A screenshot of the LatFit web interface result visualisation.

Results are visualised using Jmol [43] for an interactive presentation of the final protein structure. The final dRMSD and cRMSD values of the lattice protein compared to the original protein are given as well as the absolute move string encoding of the resulting structure. For an example of the LatFit web interface see Figure 2.

Further details regarding the methods implemented, the output formats supported and the applicable parameterisation are located in the LatFit manual distributed with the source code. We provide an extensive help page and a frequently asked questions (FAQ) section within the web interface. Note, the web server is based on JavaServer Pages (JSP) technology and requires a connection via the JSP standard port 8080. A web interface for *ad hoc* usage is available at http://cpsp.informatik.uni-freiburg.de/LatFit/  and   http://cpsp.informatik.uni-freiburg.de:8080/.

## 3. Results and Discussion

In the following, we evaluate the average fitting quality of our new LatFit tool to results known from literature [6, 8, 13]. Furthermore, we investigate the performance of the new dRMSD-based fitting procedure implemented in LatFit. To this end, we compare its results to the cRMSD-optimizing approach that follows [6, 18], both implemented within LatFit.

We use LatFit to derive protein models on the commonly used 3D cubic, FCC, and knights walk lattices [18] using the dRMSD-based approach, parameterised with *n*_keep_ = 1000. Our test set was taken from the PISCES web server [44]. We enforced 40% sequence identity cutoff, chain length 50–300, R-factor ≤0.3, and resolution ≤1.5 Å to derive a high-quality set of proteins to model. Given our requirement for side chains, *C*_*α*_-only chains were ignored. The resulting benchmark set contains 1198 proteins exhibiting a mean length of 160 (*σ* = 64).

In accordance with previous studies [18], cRMSD and dRMSD are used to assess model quality. cRMSD measures the similarity in according coordinate position of two structures whereas dRMSD measures the similarity of intramolecular distances. Due to the scaling of our lattice, RMSD results are in Å rather than the scaled values provided by Ponty et al. [22].

Our backbone model RMSD values presented in Table 1 are competitive or superior to known fitting results known from the literature [6, 13, 18]. Both the new dRMSD- as well as the reimplemented cRMSD-optimisation method reproduce the high quality previously achieved by other methods using the FCC and 210 lattices. The slightly higher mean cRMSD values for the dRMSD method are due to the nonoptimisation of that measure. Note, LatFit outperforms the results reported for LocalMov e by Ponty et al. [22]. We found the LocalMove webserver currently not working for the proteins tested. Therefore, only results reported in [22] for the 3D cubic lattice and no FCC results are available.

LatFit is designed for side chain models and results here are strong (see Table 1(b)). In general, side chain models produce slightly larger RMSD values than the equivalent backbone-only model. This is due to the fact that the variation in distance between consecutive *C*_*α*_ atoms (fitted in both models) is lower than that between *C*_*α*_ atoms and their side chain centroid (fitted only in side chain models). In lattice models every distance is fixed at 3.8 Å which results in a higher mean displacement of the side chain. Nevertheless, high accuracy fits are still attained. Results in our test set have mean dRMSDs of about 1.2 Å and 1.5 Å in the 210 and FCC lattice, respectively, for both optimisation strategies. When comparing the dRMSD optimisation with the cRMSD-optimising version, we observe very similar results. This is in accordance to our observations from the backbone-only models.

The strength of LatFit is its ability to produce both side chain and backbone-only lattice protein models. High accuracy models can be produced on the FCC lattice within seconds to minutes depending on the parameterisation. Fits on the 210 lattice take orders of magnitude longer for relatively little gain in model accuracy. For this reason we recommend using the FCC lattice for detailed high-throughput protein structure studies in both backbone-only and side chain representing lattice models.

## 4. Concluding Remarks

 LatFit enables the automated high resolution fitting of both backbone and side chain lattice protein models from full atomic data in PDB format. We demonstrate its high accuracy on three widely used lattices using a large, nonredundant protein data set of high resolution. Side chain fits show on average a higher deviation than backbone models, but both produce high quality fits with results generally less than 1.5 Å on the face-centred cubic lattice. To our knowledge, this is the first study and publicly available implementation for side chain models in this field. Available via web interface and as a stand-alone tool, LatFit addresses the lack of available programs and is well placed to enable further, more detailed investigation of protein structure in a reduced complexity environment. Even now the LatFit webserver is in daily use worldwide (monitored via Google Analytics, http://www.google.com/analytics/), which shows the need for efficient implementations such as LatFit.

## Figures and Tables

**Table 1a:** (a) Backbone-only models

	Park and Levitt [18]		Reva et al. [14, 22]	Ponty et al. [22]	LatFit	
	cRMSD	dRMSD	cRMSD^*∗*^	cRMSD^*∗*^	cRMSD	dRMSD
CUB	2.84	2.34	2.84 (0.748·3.8)	3.46 (0.911·3.8)	2.97	**2.08 **
FCC	1.78	1.46	—	—	1.89	**1.34 **
210	1.24	1.02	—	—	1.29	**0.92 **

**Table 1b:** (b) Side chain models

	LatFit	
	cRMSD	dRMSD
CUB	4.16	2.78
FCC	2.10	1.50
210	1.60	1.13
